# Cutaneous Biodistribution: A High-Resolution Methodology to Assess Bioequivalence in Topical Skin Delivery

**DOI:** 10.3390/pharmaceutics11090484

**Published:** 2019-09-18

**Authors:** Julie Quartier, Ninon Capony, Maria Lapteva, Yogeshvar N. Kalia

**Affiliations:** School of Pharmaceutical Sciences, University of Geneva, CMU-1 rue Michel Servet, 1211 Geneva 4, Switzerland; Julie.Quartier@unige.ch (J.Q.); ninoncapony@gmail.com (N.C.); maria.lapteva@unige.ch (M.L.)

**Keywords:** bioequivalence, econazole nitrate, cutaneous biodistribution profile, topical skin delivery, generics, reference medicinal product

## Abstract

A draft guideline from the European Medicines Agency (EMA) highlights the need for methods to assess the quality/equivalence of topical drug formulations. The “cutaneous biodistribution method”, which provides insight into a drug’s spatial distribution in the epidermis/dermis, was used to compare cutaneous bioavailability of econazole nitrate (ECZ) from a reference medicinal product (RMP) and two approved bioequivalent generic creams under finite dose conditions. Statistically significant differences between the ECZ biodistributions from the RMP/Generics were determined and used with acceptance criteria based on those from the EMA to evaluate bioequivalence. In porcine skin, ECZ deposition in total skin, epidermis, upper and lower dermis from Generic 1 was within the acceptance interval, contrary to Generic 2, which was marginally below it. For human skin, Generic 1 deposition was marginally above the acceptance interval and not bioequivalent. The results were consistent with those using the EMA’s acceptance intervals using the ratio of the mean ECZ depositions of Generic 1 and the RMP. Differences identified using this data-rich technique may not translate to observable differences in clinical efficacy; however, generics with non-statistically different biodistributions to the RMP should have a comparable clinical effect. The cutaneous biodistribution method could benchmark the development of topical generic products.

## 1. Introduction

The therapeutic equivalence of systemically acting drugs can be demonstrated using bioequivalence (BE) studies that compare pharmacokinetic parameters, e.g., C_max_, T_max_, and AUC, of the reference medicinal product (RMP) and the generic product in a small healthy volunteer population. The key point of these studies is to correlate the rate and extent of drug absorption *in vivo* to the efficacy and to confirm safety [1]. However, these studies are expensive, time-consuming and irrelevant for most dermatological drugs. Indeed, most topical dermatological products are intended to act locally in the skin and to limit undesirable systemic absorption. In light of this, the European Medicines Agency (EMA) proposed a guideline on the quality and equivalence of topical drugs and this was recently updated [2,3].

Currently, recommended *in vitro* assays comprise the testing of *in vitro* release (IVRT) and *in vitro* permeation (IVPT), whereas *in vivo* testing includes pharmacodynamic studies for corticosteroid products using the vasoconstriction activity of steroids to produce skin blanching at the site of application [3,4] and pharmacokinetic studies using tape stripping [3]. The major drawback of these methodologies is the lack of drug quantification in the viable epidermis and dermis that are the sites of drug action in the majority of dermatological conditions. Indeed, although the epidermis and dermis are present in skin samples used for IVPT, the bioequivalence of formulations is assessed on their ability to deliver “equivalent” amounts of drug to where it should not be: transdermal permeation of locally acting drugs and their systemic uptake *in vivo* is undesirable since it increases the risk of off-target side effects. Knowledge of the drug spatial distribution in the skin, and especially at its site of action, is clearly of great therapeutic interest and could be a useful and more relevant tool to assess bioequivalence. With the advent of highly selective and specific analytical techniques, it is now possible to quantify drug bioavailability inside the skin at the nanogramme level [5,6,7,8]. Moreover, this “cutaneous biodistribution method” enables drug delivery in the skin to be determined as a function of depth at a high resolution and the additional data therefore permit a more detailed comparison of the equivalence of different formulations to be made.

The objective of this study was to use this methodology for an “in-depth” investigation into the cutaneous delivery of econazole (ECZ) from bioequivalent products—an RMP cream and two generic products—and a non-bioequivalent solution formulation (the “negative control”; this nomenclature is taken from the EMA Guideline (Section 5.3.2: General Considerations—“*In vitro* skin permeation and *stratum corneum* sampling (tape stripping) studies should include negative controls that are not equivalent to the test and comparator products”). The topical cutaneous delivery of ECZ was determined *in vitro* after application of reference, generic and non-generic topical formulations under finite dose conditions. The principal objectives were (i) to study the accuracy, sensitivity and reproducibility of the method by analysing the biodistribution profile of ECZ from the RMP (ECZ 1% RMP cream), two approved generics (ECZ 1% Generic cream 1 and ECZ 1% Generic cream 2), and a negative control (ECZ 1% ethanolic solution) and (ii) to evaluate bioequivalence of these topical dermatological products in porcine and human skin using a modified version of the acceptance criteria given by the EMA adapted to the cutaneous biodistribution method.

## 2. Materials and Methods

### 2.1. Materials

Acetonitrile (LC-MS grade) was purchased from Fisher Scientific (Reinach, Switzerland). Formic acid (extra pure 99%) was obtained from Biosolve Chemicals (Dieuze, France). ECZ, miconazole nitrate (MCZ; analytical internal standard), isopentane and Dulbecco’s phosphate buffered saline (without calcium chloride and magnesium chloride; DPBS) was sourced from Sigma-Aldrich (Buchs, Switzerland). Brij™ C20-PA-(RB) was purchased from Croda Europe (East Yorkshire, England). O.C.T. mounting medium was obtained from VWR Chemicals (Leuven, Belgium). Ultrapure water (Millipore Milli-Q Gard 1 Purification Pack resistivity >18 MΩ·cm; Zug, Switzerland) was used to prepare all solutions. Marketed formulations of econazole—ECZ 1% RMP cream (*w*/*w*), ECZ 1% Generic cream 1 (*w*/*w*), ECZ 1% Generic cream 2 (*w*/*w*) and ECZ 1% ethanolic solution—were purchased in a local pharmacy of a Member State of the European Union.

### 2.2. Analytical Methods

Ultra-high-pressure liquid chromatography (UHPLC) coupled with tandem mass spectrometry detection was used to quantify ECZ deposited in skin during the *in vitro* delivery experiments. The UHPLC-MS/MS system consisted of a Waters Acquity UPLC^®^ system (Baden-Dättwil, Switzerland) with a binary solvent pump and sample manager and a Waters XEVO^®^ TQ-MS detector (Baden-Dättwil, Switzerland). Isocratic separation was performed using a Waters XBridge^®^ BEH column (C8, 2.1 × 50 mm, 2.5 µm). The column was thermostatted at 30 °C. The mobile phase consisted of (a) acetonitrile + 0.1% formic acid and (b) Milli-Q water + 0.1% formic acid (70:30 *v*/*v*). The flow rate was set at 0.2 mL/min and the injection volume was 5 µL. To account for the matrix effect, each injected sample contained MCZ (internal standard) at a concentration of 50 ng/mL. Tandem mass spectrometry was performed using electrospray ionization in positive mode and multiple reaction monitoring (MRM). MassLynx software was used for data integration and analysis. The UHPLC-MS/MS method was validated according to ICH guidelines (complete details are provided in the Supplementary Materials). The MS/MS settings for both ECZ and MCZ are presented in Table 1.

### 2.3. Evaluation of ECZ Skin Delivery In Vitro

#### 2.3.1. Skin Preparation

Porcine ear skin was used for the *in vitro* studies and was supplied by a local abattoir (CARRE; Rolle, CH). Briefly, skin samples were processed with a Zimmer air dermatome (Münsingen, Switzerland) to obtain samples with a thickness of ∼800 µm. Hair was removed carefully from the skin surface using clippers. The excised skin samples were then punched out (Berg & Schmid HK 500; Urdorf, Switzerland) into 30-mm diameter circular discs and stored at −20 °C until use (for a maximum period of 3 months).

Human skin samples were collected immediately after surgery from the Department of Plastic, Aesthetic and Reconstructive Surgery, Geneva University Hospital (Geneva, Switzerland). The study was approved by the Central Committee for Ethics in Research (CER: 08–150 (NAC08-051) 27/10/2008); Geneva University Hospital). The hypodermis and fatty tissue were carefully removed. The excised skin samples were then punched out into 32 mm diameter circular discs and were subsequently horizontally sliced with a Thomas Stadie-Riggs slicer (Thomas Scientific; Swedesboro, NJ, USA) to a thickness of ∼1100 µm. The skin was stored in a biobank at −20 °C (for a maximum period of 3 months).

#### 2.3.2. ECZ Delivery Under Finite Dose Conditions

Porcine skin samples were mounted in Franz diffusion cells (Millan SA; Meyrin, Switzerland) with a formulation application area of 2 cm^2^. The receiver compartment was filled with 10 mL of phosphate buffered saline at pH 7.4 containing 0.1% Brij™ C20 to maintain sink conditions. The formulation under investigation (10 ± 0.5 mg/cm^2^, corresponding to 100 µg of ECZ per cm^2^) was applied to the *stratum corneum* surface in the donor compartment and left in contact with the skin sample for 12 h. The receptor compartment was stirred at 250 rpm and maintained at 32 °C throughout the experiment. At the end of the experiment, 1 mL of the receptor compartment was collected to quantify ECZ permeation. Each skin sample was carefully cleaned with cotton swab and a wash solution to remove the remained formulation from skin surface. These conditions comply with the OECD guidelines [9].

#### 2.3.3. Investigation of ECZ Biodistribution Profile

At the end of the experiment, a small area of 0.8 cm^2^ was punched out from the 2 cm^2^ skin samples. These skin discs were snap-frozen in isopentane cooled by liquid nitrogen. For this step, the skin samples were fixed with O.C.T. on a round cork and a plastic o-ring was carefully placed around the skin discs to avoid tissue compression and to ensure a flat frozen sample. This technique ensured that the integrity of the thickness of the different regions of the skin was guaranteed. The skin discs were then cryotomed (Thermo Scientific CryoStar™ NX70; Reinach, Switzerland) to obtain 2 lamellae with a thickness of 20 μm and 19 lamellae with a thickness of 40 μm; these 21 lamellae (2 × 20 µm + 19 × 40 µm) enabled the amounts of ECZ to be determined as a function of position down to a depth of ~800 μm, encompassing the *stratum corneum*, viable epidermis and upper dermis, respectively. ECZ deposited in each lamella was extracted in 500 µL of acetonitrile: Milli-Q water (80:20, *v*/*v*) overnight with continuous stirring at room temperature. The samples were centrifuged at 5000 rpm for 10 min and diluted prior to UHPLC-MS/MS analysis.

### 2.4. Data Analysis

Data were expressed as the mean ± confidence interval 90% (CI_90%_). Outliers determined using the Grubb’s test were discarded.

#### 2.4.1. Biodistribution Profile

The results were evaluated using acceptance criteria implemented especially for this study. The recommendations given by the EMA to assess bioequivalence were modified and simplified in order to better evaluate the large amount of data generated by the cutaneous biodistribution method. As in the EMA recommendations, “narrow acceptance criteria” (acceptable interval of 80.00–125.00%) and “wide acceptance criteria” (acceptable interval of 69.84–143.19%) were defined [3]. However, for this method, intervals of acceptance criteria were calculated based on the mean of ECZ deposition from the RMP using Equations (1) and (2):

(1) $${“\mathrm{narrow}\;\mathrm{acceptance}\;\mathrm{criteria}”\;=\;[\mathrm{ECZ}}_{\mathrm{RMP}\;\mathrm{deposition}}\;\times \;0.8;\;{\mathrm{ECZ}}_{\mathrm{RMP}\;\mathrm{deposition}}\;\times \;1.25]$$

(2) $${“\mathrm{wide}\;\mathrm{acceptance}\;\mathrm{criteria}”\;=\;[\mathrm{ECZ}}_{\mathrm{RMP}\;\mathrm{deposition}}\;\times \;0.6984;\;{\mathrm{ECZ}}_{\mathrm{RMP}\;\mathrm{deposition}}\;\times \;1.4319]$$

The mean ± CI_90%_ of the amount of ECZ deposited from the tested generic product (either that within each lamella, or in the epidermis, upper and lower dermis or the total amount in the skin) needed to be within the acceptance interval (narrow or wide) to be considered bioequivalent to the RMP.

#### 2.4.2. Statistical Analysis

The results were also compared statistically using a Student’s *t* test. The level of significance was fixed at *α* = 0.05.

#### 2.4.3. Bioequivalence Assessment by Ratio of Means

The results obtained with ECZ 1% Generic cream 1 (using both porcine and human skin) were also evaluated using the bioequivalence assessment recommended by the EMA as stated in the draft guideline, “The 90% confidence interval for the ratio of means of the test and comparator products should be contained within the acceptance interval” [3]. For this purpose, the ratio of the mean ECZ deposition in each lamella, each anatomical regions of the skin (epidermis, upper and lower dermis), and in total skin, of the generic product and the RMP was determined (3).

(3) $$\mathrm{Ratio}\;\mathrm{of}\;\mathrm{means}\;=\;\frac{{\mathrm{Mean}\;\mathrm{ECZ}}_{\mathrm{Generic}\;\mathrm{deposition}}}{{\mathrm{Mean}\;\mathrm{ECZ}}_{\mathrm{RMP}\;\mathrm{deposition}}}$$

CI_90%_ values for the ratio of means were calculated with Fieller’s theorem using GraphPad software [10]. A wide acceptance interval of 69.84–143.19% was used.

## 3. Results and Discussion

### 3.1. Biodistribution and Evaluation of Bioequivalence of ECZ Products in Porcine Skin

#### 3.1.1. Validation of the Methodology

New methodologies to assess bioequivalence should exhibit sensitivity, selectivity, accuracy and reproducibility [2]. The sensitivity and accuracy of the methodology was ensured by the use of UHPLC-MS/MS to quantify ECZ: the analytical method was validated and the limits of quantification in skin matrix and in the receptor compartment medium (PBS + 0.1 Brij™ C20 solution) were 2.0 and 10.0 ng/mL, respectively. No ECZ was detected in blank skin samples.

**Figure 1:**
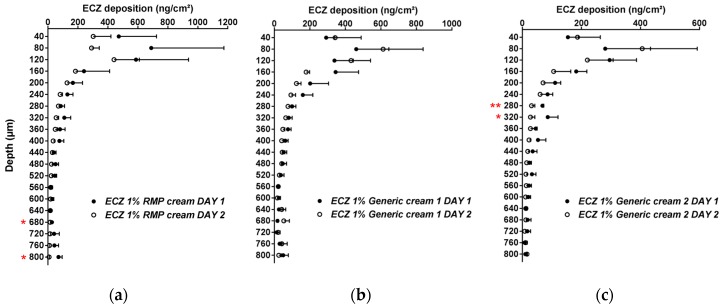
Inter-day reproducibility of the cutaneous biodistribution profile of ECZ in porcine skin lamellae (2 × 20 µm + 19 × 40 µm) to 800 µm (full-depth) for the three formulations: (**a**) reference medical product (ECZ 1% reference medicinal product (RMP) cream), (**b**) ECZ 1% Generic cream 1 and (**c**) ECZ 1% Generic cream 2. Tested groups: Day 1 (●) and Day 2 (○). (Mean ± CI_90%_; *n* = 6). *p*-values were calculated using a Student’s *t* test; statistically significant differences are denoted by asterisks (* *p* < 0.05; ** *p* < 0.005).

The inter-day reproducibility of the method was also evaluated and, aside from two skin lamellae for ECZ 1% RMP cream and Generic cream 2, there were no statistically significant differences in the biodistribution profile of ECZ for the three formulations tested on two separate days (*p* > 0.05, *n* = 6) (Figure 1).

**Figure 2:**
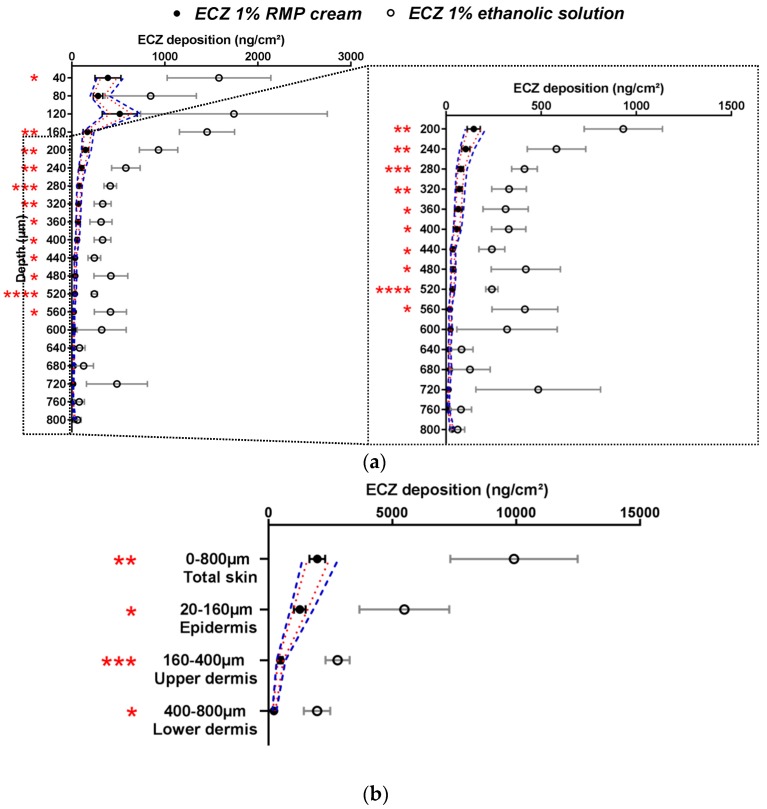
Cutaneous biodistribution profile of ECZ: (**a**) in porcine skin lamellae (2 × 20 µm + 19 × 40 µm) to 800 µm (full depth) and (**b**) as a function of position in the anatomical regions of the skin (epidermis, upper and lower dermis) and in total skin. Tested groups: reference medical product (ECZ 1% RMP cream) (●) and non-bioequivalent “negative control” (ECZ 1% ethanolic solution) (○). (Mean ± CI_90%_; *n* = 12 and *n* = 6, respectively). Intervals of acceptance criteria were calculated based on the mean ECZ deposition from the RMP; (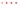) interval of 80.00–125.00% and (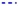) interval of 69.84–143.19%. *P*-values were calculated using a Student’s *t* test; statistically significant differences are denoted by asterisks (* *p* < 0.05; ** *p* < 0.005; *** *p* < 0.0005; **** *p* < 0.00005).

The selectivity of the methodology was proven by comparing the RMP and the negative control (a non-generic and hence non-bioequivalent formulation—ECZ 1% ethanolic solution). The approved ethanolic solution of ECZ was chosen as the negative control because it was considered that the dosage form would strongly affect ECZ deposition as compared to the ECZ 1% RMP cream. ECZ skin distribution from this solution and the RMP are shown in Figure 2.

ECZ deposition from the ECZ solution was significantly higher than from the RMP in the majority of skin lamellae in all the major layers of the skin (epidermis, upper and lower dermis) and in total skin. Considering the acceptance criteria (both narrow (1) and wide (2)), the ECZ 1% solution was indeed non-bioequivalent and this can be explained by the presence of ethanol: a well-known penetration enhancer [11].

Moreover, ECZ permeation after application of the ethanolic solution was 215.0 ± 54.4 ng/cm^2^, whereas for the RMP the amount of ECZ in the receptor compartment was below the LOQ (<10 ng/mL). Thus, it was demonstrated that the method was selective and able to detect differences between formulations with the same drug content.

#### 3.1.2. Width of the Acceptance Criteria

The acceptance criteria that were used to test for bioequivalence were based on the EMA’s recommendations on *in vitro* skin permeation studies (IVPT), which permits a 69.84–143.19% interval for highly variable drug products [3]. Drug products are considered as highly variable when intra-subject variability for a parameter is larger than 30% [12]. For the acceptance criteria implemented for the cutaneous biodistribution method, the same criteria were considered. Since the variability of ECZ deposition in porcine skin was above 30%, and even above 50% for several skin layers, for the three formulations (Table 2), the wide acceptance interval of 69.84–143.19% was selected for the subsequent experiments to assess bioequivalence of ECZ products with the RMP.

#### 3.1.3. Comparison of the ECZ Biodistribution from Two Generic Drug Products and the RMP

The ECZ biodistribution in porcine skin after application of the RMP cream and Generic creams 1 and 2 for 12 h was compared by quantifying ECZ deposition in each lamella (2 × 20 µm + 19 × 40 µm) and by comparing the amounts in the major skin layers (epidermis, upper and lower dermis) as well as with respect to the total amount in the skin (experiments were performed using 12 replicates).

**Figure 3:**
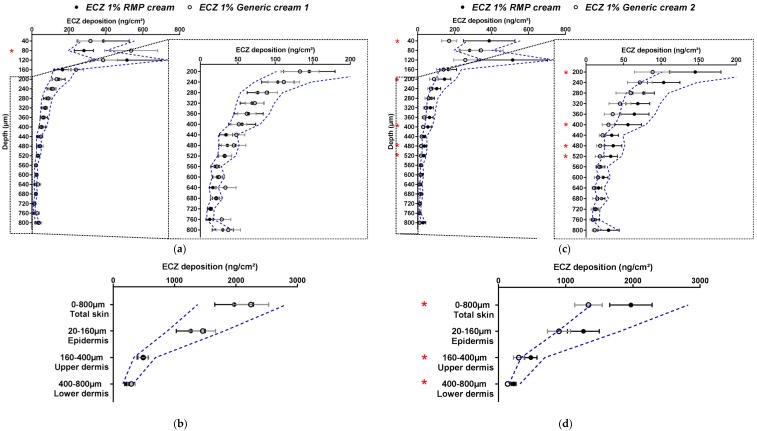
Cutaneous biodistribution profile of ECZ: (**a**) and (**c**) in porcine skin lamellae (2 × 20 µm + 19 × 40 µm) to 800 µm (full-depth) and (**b**) and (**d**) as a function of position in the anatomical regions of the skin (epidermis, upper and lower dermis) and in total skin. Tested groups: reference medical product (ECZ 1% RMP cream) (●) and generic products (ECZ 1% Generic cream 1 and ECZ 1% Generic cream 2) (○). (Mean ± CI_90%_; *n* = 12). Intervals of acceptance criteria were calculated based on the mean ECZ deposition from the RMP; (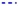) interval of 69.84–143.19%. *P*-values were calculated using Student’s *t* test; statistically significant differences are denoted by asterisks (* *p* < 0.05).

The first generic (ECZ 1% Generic cream 1) showed almost no statistically significant differences (Student’s *t*-test) to the RMP in terms of the biodistribution profile. However, the ECZ deposition of the generic was not always within the wide acceptance interval of 69.84–143.19% for all of the skin lamellae, especially those for the uppermost and deepest layers (80, 120, 160 and 440, 480, 640, 760 and 800 µm) (Figure 3a). In contrast, when the results were presented in terms of ECZ deposition in the major anatomical layers of the skin (epidermis 20–160 µm; upper dermis 160–400 µm; lower dermis 400–800 µm) and in total skin, then the drug bioavailability of the generic, ECZ 1% Generic cream 1, was within the acceptance criteria for all skin layers and in total skin (Figure 3b). Thus, the data-rich nature of the detailed biodistribution profile was perhaps too discriminating and identified punctual statistically significant differences in the amounts present at different depths that were masked when these same data points were grouped together to define discrete skin regions.

For ECZ 1% Generic cream 2, the biodistribution profile showed more numerous statistical differences (Student’s *t*-test) to the RMP than ECZ 1% Generic cream 1. When considering the wide acceptance criteria, ECZ deposition from Generic cream 2 was marginally outside the acceptance interval for almost all of the skin lamellae—being slightly below the lower limit (Figure 3c). The same observation was made when ECZ bioavailability in total skin, epidermis, upper and lower dermis were studied (Figure 3d). This reflects the stringent nature of even the wide acceptance criteria when used in the assessment of topical drug delivery given the small amounts of drug present in the skin following the use of finite dose conditions and the intrinsic variability. For the three formulations, the amount of ECZ in the receptor compartment was below the LOQ (in contrast, to the non-bioequivalent ECZ 1% ethanolic solution).

Based on the different information that can be provided by these results, Table 3 summarizes the bioequivalence assessment that can be proposed according to the acceptance criteria used for this method. The biodistribution profile and the quantification of drug amounts present in each skin lamella gave a multiplicity of extremely detailed information, making comparison and evaluation of whether the two generic creams were “bioequivalent” to the RMP even more stringent. It is worth noting that the upper lamellae of the skin biodistribution profile are subject to higher variability due to skin surface irregularity and the presence of skin appendages. On the other hand, the expression of the results as drug deposition in total skin and individual skin layers, collapsed the number of data points and resulted in less overall variability facilitating the assessment of “bioequivalence”: ECZ 1% Generic cream 1 could now be consistently judged bioequivalent to the RMP using both assessment methods, but ECZ 1% Generic cream 2 still could not. From a clinical (and regulatory) perspective, the evaluation of the drug delivery to the skin layers corresponding to its site of therapeutic action might be more relevant than the studies of drug deposition, lamellae by lamellae throughout the entire skin sample.

The selectivity of the methodology and its ability to discriminate a non-bioequivalent formulation was demonstrated with the experiment using ECZ 1% ethanolic solution, meaning that changes in the composition of the product can be detected by the cutaneous biodistribution profile. In the case of the three ECZ creams, the qualitative composition of the RMP is close to the generic products, regarding the content in emulsifier, surfactants, stabilizer, solubilizing and dispersant agents [13]. However, the quantitative proportion of these excipients might differ (not detailed by the manufacturer). As the formulation can affect the thermodynamic activity of the drug or skin permeability, even small changes in the composition could influence drug release and its deposition in the skin [14,15]. This could explain the difference between the two generic creams for the assessment of topical bioequivalence to the RMP.

### 3.2. Biodistribution and Evaluation of Bioequivalence of ECZ Products in Human Skin

**Figure 4:**
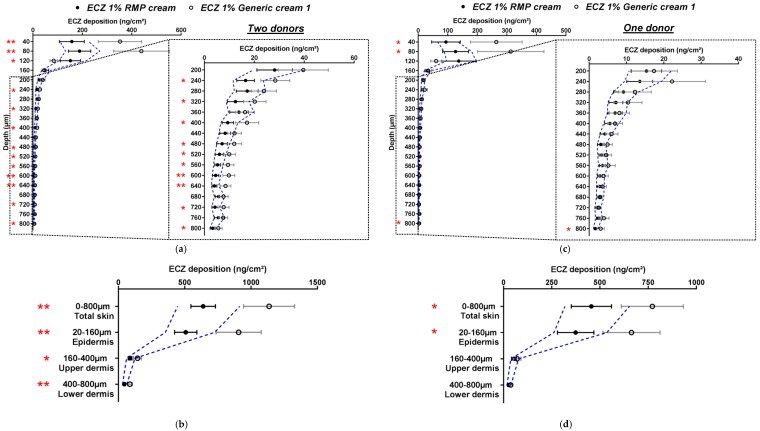
Cutaneous biodistribution profile of ECZ: (**a**,**c**) in human skin lamellae (2 × 20 µm + 19 × 40 µm, to 800 µm (full-depth) and (**b**,**d**) as a function of position in the anatomical regions of the skin (epidermis, upper and lower dermis) and in total skin; Tested groups: reference medical product (ECZ 1% RMP cream) (●) and generic product (ECZ 1% Generic cream 1) (○). (Mean ± CI_90%_; *n* = 24 from two donors for (**a**,**b**) and *n* = 12 from one donor for (**c**,**d**)). Intervals of acceptance criteria were calculated based on the mean ECZ deposition from the RMP; (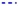) interval of 69.84–143.19%. *p*-values were calculated using Student’s *t* test analysis; statistically significant differences are denoted by asterisks (* *p* < 0.05; ** *p* < 0.005).

In the next part of the study and in accordance with EMA recommendations for *in vitro* permeation studies, human skin was used for the next set of experiments and the number of replicates was increased to 24 [3]. Given the results obtained with porcine skin, Generic cream 1 was selected for these studies. The bioequivalence of ECZ 1% Generic cream 1 with the ECZ 1% RMP cream was evaluated using 24 skin pieces (replicates) originating from two different donors. As for studies with porcine skin, the variability of ECZ deposition in human skin was more than 30% for both formulations; hence, the wide acceptance criteria of 69.84–143.19% was used to compare the generic with the RMP (Figure 4a,b).

It was noted that the absolute amounts deposited in the different layers in human skin were lower than those observed with the porcine surrogate (ECZ total skin deposition from ECZ 1% RMP cream was 1971.1 ± 315.1 and 638.6 ± 92.52 ng/cm^2^ in porcine and human skin, respectively, and from ECZ 1% Generic cream, 2243.6 ± 290.7 and 1135.4 ± 192.14 ng/cm^2^, respectively). Moreover, the biodistribution profile of ECZ showed that drug deposition from the generic formulation was no longer contained within the wide acceptance interval. The amounts of ECZ deposited after application of Generic cream 1 were systematically higher. The same observation was made for ECZ deposition in total skin and each of the skin compartments (epidermis, upper and lower dermis) (Figure 4b).

The cutaneous biodistribution profile of ECZ for both formulations was then evaluated with human skin samples originating from the same donor (Figure 4c,d). Despite the fact that the studies were performed with human skin originating from one donor, ECZ 1% Generic cream 1 could still not be considered bioequivalent to ECZ 1% RMP cream, based on the entire biodistribution profile and on delivery to the total skin and the epidermis using the wide acceptance criteria. The results demonstrated that the “non-bioequivalence” of ECZ 1% Generic cream 1 to RMP was not related to the donor origin. For both formulations, the amount of ECZ in the receptor compartment was below the LOQ. In conclusion and contrary to the preliminary studies performed with porcine skin, biodistribution profile of ECZ from ECZ 1% Generic cream 1 in human skin could not be considered as bioequivalent to the RMP. It is well-known and accepted that porcine skin can be used as surrogate for human skin for *in vitro* experiments [16,17]. However, it is possible that, despite the anatomical similarities between porcine and human skin, some variations in skin structure influence skin permeability. Indeed, porcine ears have a higher density of hair follicles, which is known to increase the drug penetration rate [18,19]. 

Furthermore, the increase of drug deposition in porcine skin compared to human skin has already been observed in other topical delivery studies and the extent of any difference may also depend on the type of formulation [5,7]. This could explain why the method, performed with porcine skin, was less discriminating than the method performed with human skin to assess bioequivalence of ECZ 1% Generic cream 1.

It is worth noting that Generic cream 1 has been approved as being bioequivalent to RMP by the competent national agencies, presumably based on clinical comparisons. This would mean that the quantifiable statistically significant difference between the RMP and Generic cream 1 with respect to drug deposition in the skin (and using the wide acceptance criteria) was evidently not significant enough to produce a clinically relevant difference. However, the reverse is not necessarily true: if a generic topical product can demonstrate that it is able to deliver the same amounts of drug in every skin lamella as the RMP, i.e., have a statistically equivalent biodistribution profile, then the clinical effect should be equivalent to that of the RMP since there is no statistically significant difference between the amounts of drug substance present.

### 3.3. General Evaluation of the Methodology

#### 3.3.1. Comparison with the Other Accepted/Promising Methods

The advantages and limitations of this method as compared to existing methods to assess bioequivalence are presented in Table 4. The major advantage of the proposed methodology is that the drug quantification enables an assessment of topical bioavailability based on the drug concentration in the target skin layer (i.e., efficacy) and drug concentration at other sites (i.e., safety). This exact quantification and localization of the drug as a function of depth in the different skin regions cannot be achieved with other methods. The analytical method used here (UHPLC-ESI-MS/MS) enables the specific and quantitative analysis of a very broad panel of molecules (small and high molecular weight compounds, polar and less polar compounds) [20,21,22]. Moreover, as for the *in vitro* release testing, the biodistribution profile enables changes in the drug dermatopharmacokinetics to be related to modifications in the composition of the formulation. However, the cutaneous biodistribution method may encounter some limitations, especially for the assessment of bioequivalence in the SC. Indeed, this pilot study describes an additional and complementary assessment technique to the previously published tape-stripping protocols. In the particular case of ECZ, the combination of this technique with tape-stripping could be of great interest since tape stripping would assess drug delivery to the SC (target layer) and the cutaneous biodistribution technique would directly assess the off-target delivery.

#### 3.3.2. Statistical Analysis and Acceptance Criteria

These preliminary results showed that cutaneous biodistribution profiles give a unique insight into the spatial distribution of the drug in the skin by quantifying drug concentrations in the different lamellae to create a high-resolution profile of drug amounts as a function of position. However, the data-rich nature of the biodistribution profile and the presence of multiple data points from the same anatomical region increases the probability that the assessment of bioequivalence of a generic product can differ from one layer to another in the same skin region, when it comes to considering the acceptance criteria (Figure 3a). Reducing the number of data points decreases this risk and it might be argued that it is more appropriate to assess the bioequivalence or the non-bioequivalence of the product by comparing drug deposition in the major regions of the skin.

In general, the results obtained with porcine and human skin highlight the strictness of the acceptance criteria established for this method. When pairwise Student *t*-tests were applied, it was observed that ECZ deposition from a generic product (for example ECZ 1% Generic cream 1) in certain lamellae could be considered as not being statistically significantly different to the RMP (*p* > 0.05) despite the fact that it was not within the acceptable criteria range (Figure 3a). 

The major reason for these two different interpretations is the large CI_90%_, especially for studies with porcine tissue. However, when the number of replicates was increased to 24 during human skin studies, the CI_90%_ for ECZ deposition decreased and the conclusions given by Student *t*-tests and acceptance criteria about the bioequivalence of ECZ 1% Generic cream 1 with the RMP mostly correlated.

**Figure 5:**
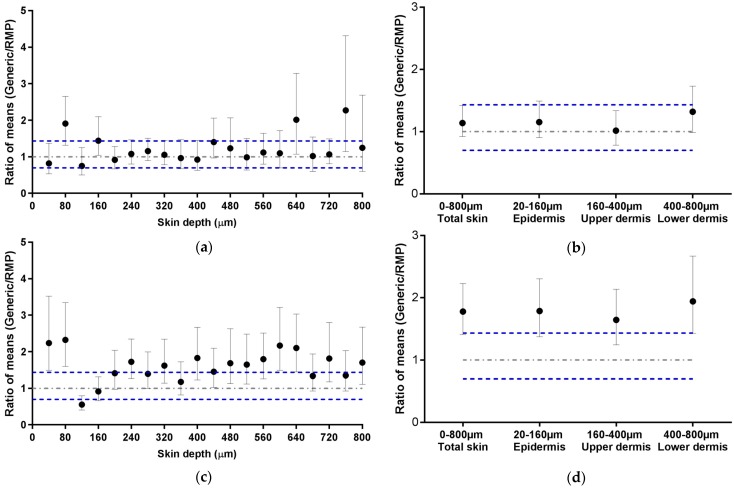
Ratio of mean ECZ depositions from the ECZ 1% Generic cream 1 and the RMP (●): (**a**) in porcine skin lamellae (2 × 20 µm + 19 × 40 µm to 800 µm (full-depth) and (**b**) as a function of position in the anatomical regions of the skin (epidermis, upper and lower dermis) and in total skin, (**c**) in human skin lamellae (2 × 20 µm + 19 × 40 µm), from 2 donors, to 800 µm (full-depth), and (**d**) as a function of position in the anatomical regions of the skin (epidermis, upper and lower dermis) and in total skin. (Ratio ± CI_90%_; *n* = 12 for (**a**,**b**) and *n* = 24 for (**c**,**d**)). Intervals of acceptance criteria were chosen according to the EMA guidelines; (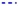) interval of 0.6984–1.4319.

The acceptance criteria implemented for this study are different but closely related to those given by the EMA in their recommendations for *in vitro* permeation studies (Section 2.4.3). The EMA has defined that the ratio of the means of “equivalence parameters” of the generic and the RMP should be contained within the acceptable interval of 0.8–1.25 or 0.6984–1.4319 for highly variable drugs products. Figure 5 presents the ECZ deposition results expressed as a ratio of means ± CI_90%_ for the Generic:RMP. This representation is consistent with the biodistribution profile representations (Figure 3 and Figure 4) since data points lying outside of the acceptance interval for instance in Figure 3a also fall outside the interval in Figure 5a. It appears that the CI_90%_ are slightly increased by using the representation of the ratio of the means since ECZ deposition in porcine epidermis in Figure 3b was within the acceptance range, whereas in Figure 5b, the CI_90%_ marginally intercepts the upper acceptance limit. Human skin deposition results are also consistent (Figure 4a,b and Figure 5c,d). Overall, it can be concluded that both data representations are consistent, thus confirming that, based on the biodistribution data, ECZ 1% Generic cream 1 can be judged equivalent in porcine skin but not in human skin.

These results also highlight the stringency of the acceptance criteria for *in vitro* skin delivery studies and that the choice of the range should balance the presence of statistically significant differences, which may involve nanogramme quantities of drug, with the therapeutic/clinical relevance. In other words, the acceptance range should be set considering the criteria of efficacy and safety for drug bioavailability in the skin. Further studies with other marketed generic products of different APIs are currently being performed in that sense. The objective of these studies is to use already approved bioequivalent products to fine-tune the biodistribution method and to define standard parameters, e.g., acceptance criteria, within which the mean drug deposition and its CI_90%_ are contained, and thus assess the bioequivalence of topical generic products with the RMP. Given the differences between the results obtained with porcine and human skin, other molecules are currently being tested to establish a correlation between drug bioavailability in these two skin tissues.

## 4. Conclusions

These studies show the amount of information on the spatial distribution of drugs in the skin that can be obtained with this new methodology. The combination of a robust cryotoming method and the highly sensitive UHPLC-MS/MS technique gives quantitative information about the amounts of drug deposited in the major regions of the skin and also enables a precise profile of drug localization in the skin to be determined. The studies described here were at a resolution of 40 µm (higher resolution, i.e., to 20 µm is also possible [5]). Since the “cutaneous biodistribution method” enables a detailed comparison of the ability of formulations to deliver a drug into the skin, it follows that there should be potential applications in the evaluation of bioequivalence. The biodistribution profile of the RMP could be used as a tool to benchmark the equivalence of new generic formulations during pharmaceutical product development. The equivalence would be based on the drug amounts actually present in the skin, the target compartment, and not on “undesirable” and off-target transdermal permeation. In theory, generic formulations that displayed no statistically significant difference to the RMP and ideally, were within the limits of the acceptance criteria, thereby delivering equivalent amounts of drug to the different regions in the skin, should be bioequivalent. This methodology would represent a cost and time-saving complement to IVRT and IVPT studies and choose the best candidate for further *in vivo* studies. The method could also be of significant support when there are modifications to the excipients in the formulations. However, the data rich nature of the technique is a “double-edged sword” in that comparison of the multiplicity of data points i.e., the respective quantities in the corresponding lamellae create more opportunities for specific data point pairs to display statistically significant differences. Regrouping these data points to the respective anatomical regions and/or the total skin deposition enables these differences to be masked facilitating statistical comparison but at the expense of the high-resolution analysis of the biodistribution. Therefore, further work is required to develop an appropriate statistical framework that enables the data-richness of the technique to be fully exploited; this will be done after consultation with the regulatory authorities.

This work did not question the bioequivalence of the two generic products of ECZ studied, as these formulations have already been assessed bioequivalent and approved by the authorities (presumably after clinical trials) but it might be considered as showing that quantifiable, statistically significant differences in drug bioavailability may not translate to differences that are clinically significant when it comes to patient therapy. Further work is ongoing to increase the number of replicates for the ECZ study and to investigate the delivery of other APIs and types of formulation.

## Figures and Tables

**Table 1:** MS/MS Setting for detection of econazole nitrate (ECZ) and miconazole nitrate (MCZ).

Parameter	Econazole	Miconazole
Nature of parent ion	[M + H]^+^	[M + H]^+^
Parent ion (*m*/*z*)	381.1	417.1
Daughter ion (*m*/*z*)	124.9	158.9
Collision energy (V)	36	34
Cone voltage (V)	36	46
Capillary voltage (kV)	3.10	3.10
Capillary temperature (°C)	350	350
Desolvation gas flow (L/h)	650	650
Cone gas flow (L/h)	3	3
Collision gas flow (L)	0.15	0.15
LM resolution 1	2.96	2.96
HM resolution 1	15	15
Ion energy 1 (V)	0.3	0.3
LM resolution 2	2.91	2.91
HM resolution 2	15.24	15.24
Ion energy 2 (V)	0.6	0.6

**Table 2:** Variability of ECZ deposition in the different porcine skin layers for each formulation presented as % RSD values (*n* = 12).

Skin Layers	ECZ 1% RMP Cream	ECZ 1% Generic Cream 1	ECZ 1% Generic Cream 2
Total skin	60%	53%	64%
Epidermis	68%	48%	70%
Upper dermis	48%	37%	59%
Lower dermis	61%	63%	63%

**Table 3:** Summary of bioequivalence assessment based on different information extracted from the results (wide acceptance criteria).

Assessment of Topical Bioequivalence to RMP	ECZ 1% Generic Cream 1	ECZ 1% Generic Cream 2
Based on the high resolution biodistribution profile	Non-equivalent	Non-equivalent
Based on the total skin delivery	Equivalent	Non-equivalent
Based on delivery to individual skin layers	Equivalent	Non-equivalent

**Table 4:** Advantages and limitations of accepted/promising method and the proposed methodology to assess bioequivalence (adapted with permission from Patel et al. [23]. Copyright Elsevier, 2016.) [23,24,25,26].

Technique	*In Vitro/Ex Vivo*	*In Vivo*	Advantages	Limitations
IVRT *in vitro* release testing	X		Provides essential data during optimization process of formulation, production process, post-marketing changes and quality control Sensitive and discriminatory power to microstructure and drug content changes Allows high throughput screening Time and cost-efficient	Limited correlation with *in vivo* delivery
IVPT *in vitro* permeation testing	X		Uses available animal skin model or excised human skin Integral part of the quality control of transdermal drug systems Allows high throughput screening Time and cost-efficient	Suffers from animal and human skin variability Limited to the study of skin-permeating compounds Does not allow consideration of *in vivo* skin processes like blood circulation/metabolism Comparison based on “off-target” testing
Tape stripping/dermatopharmacokinetics	X	X	Minimally invasive Sensitive to drug clearance from SC, thus allows kinetic measurements Applicable for diseased skin Cost-efficient	Only valid for drugs whose target organ is SC Suffers from inter-subject and inter-laboratory variabilities Time-consuming
Vasoconstriction assay		X	Can indicate drug efficacy - pharmacodynamic endpoint Allows consideration of *in vivo* skin processes like blood circulation/metabolism Applicable for diseased skin	Limited to drugs that induce local vasoconstriction High subject-to-subject variability Costly
Microdialysis/microperfusion *		X	Provides real time measurement of the rate and extent of drug penetration into the skin Allows consideration of *in vivo* skin processes like blood circulation/metabolism Applicable for diseased skin	Invasive Experiments duration limited at 8–10 h Poor reproducibility of probe insertion and manufacturing Costly
Spectroscopic techniques *	X	X	Nondestructive and noninvasive Broad range of application derived from the ability of these methods Allows consideration of *in vivo* skin processes like blood circulation/metabolism Provides exact location of drug in various skin depths Applicable for diseased skin	Need of distinct spectral peaks with sufficient intensity for the molecule studied Semi-quantitative method Long acquisition times to interpret the results Sensitive to skin heterogeneity (hydration, microbial growths)
Cutaneous biodistribution method	X		Uses available animal skin model or excised human skin Provides exact location and amount of drug in various skin depths Provides essential data during optimization process of formulation, production process, post-marketing changes and quality control Sensitive to microstructure and drug content changes Gives additional information to IVPT studies, especially if the amount of permeated drug is below the LOQ Allows high throughput screening Time and cost-efficient	Poor assessment in SC Does not allow consideration of *in vivo* skin processes like blood circulation/metabolism

* Is not part of recommended methods in EMA draft guideline.

## Supplementary Materials

The following are available online at https://www.mdpi.com/1999-4923/11/9/484/s1, Figure S1: Respective MRM traces of (**a**) ECZ standard alone (**b**) MCZ standard alone and (**c**) mixture of both compounds, Figure S2: MRM monitoring for ECZ in skin sample (381.1 → 124.9 transition) chromatogram of ECZ standard (2 ng/mL), blank extraction sample and blank permeation sample, Table S1: Intra-day precision and accuracy for quantification of ECZ in skin sample (values are given as mean ± SD), Table S2: Inter-day precision and accuracy for quantification of ECZ in skin sample (values are given as mean ± SD), Table S3: Table of abbreviations.
