# Supplementary material for: Cutaneous Biodistribution: A High-Resolution Methodology to Assess Bioequivalence in Topical Skin Delivery
Source: Pharmaceutics. 2019 Sep 18;11(9):484. doi: 10.3390/pharmaceutics11090484 (PMC6781275; doi:10.3390/pharmaceutics11090484)
Supplement: Supplementary file 1 [file pharmaceutics-11-00484-s001.pdf]

pharmaceutics

Supplementary Materials: Cutaneous Biodistribution: A High-Resolution Methodology to Assess Bioequivalence in Topical Skin Delivery

Julie Quartier, Ninon Capony, Maria Lapteva and Yogeshvar N. Kalia

Validation of the UHPLC-MS/MS Method

Specificity in Skin Matrix

Figure S1 shows the MRM trace of (a) ECZ standard alone, (b) MCZ standard alone and (c) a mixture of both compounds.

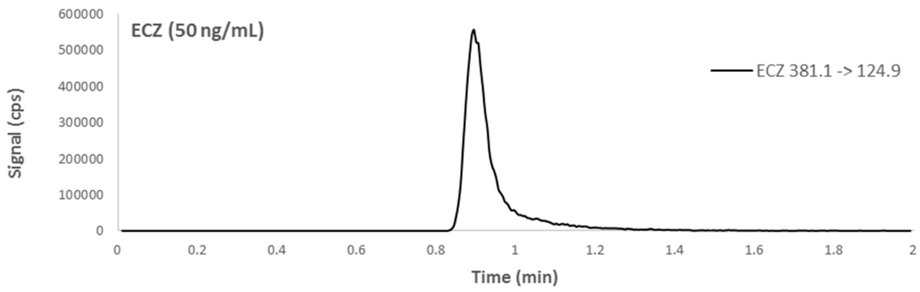

(**a**)

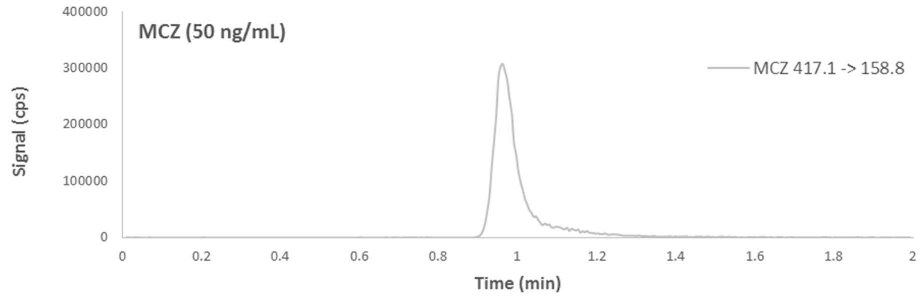

(**b**)

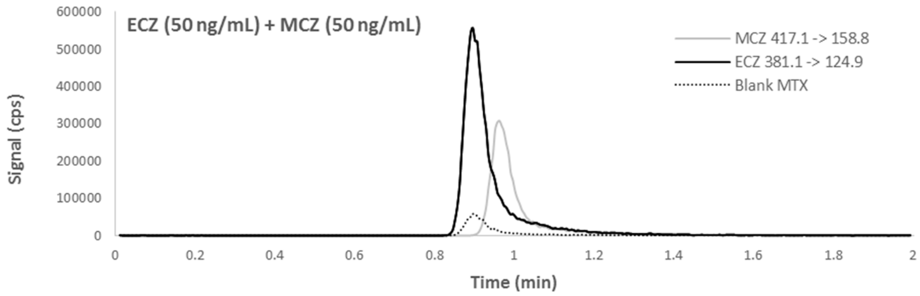

(**c**)

Figure S1. Respective MRM traces of (**a**) ECZ standard alone (**b**) MCZ standard alone and (**c**) mixture of both compounds.

Figure S2 presents the chromatogram obtained for an ECZ standard at a concentration of 2 ng/mL, a blank skin extraction sample and a blank permeation sample. No ECZ was found in the permeation sample or in the skin sample. The method was considered as specific for ECZ quantification in skin samples.

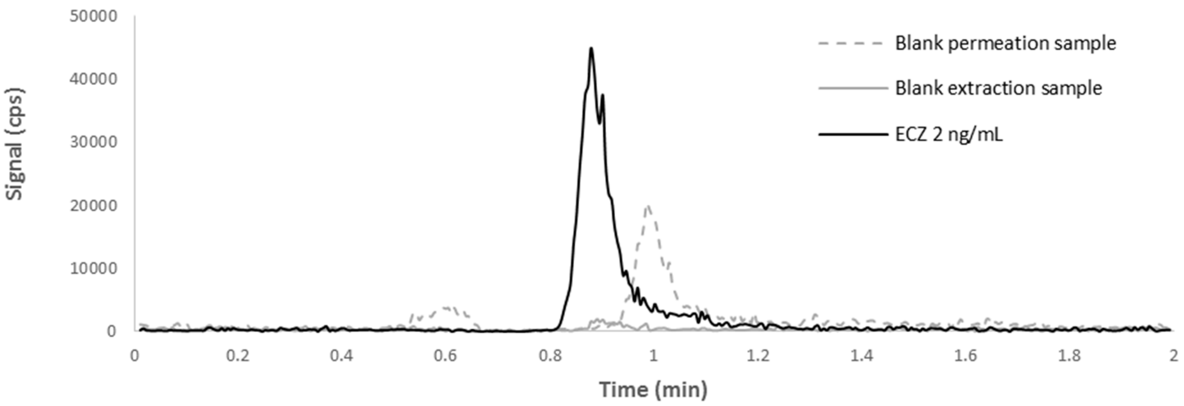

Figure S2. MRM monitoring for ECZ in skin sample (381.1 → 124.9 transition) chromatogram of ECZ standard (2 ng/mL), blank extraction sample and blank permeation sample.

Linearity

Calibration curves were constructed by plotting the ratio of ECZ and MCZ peak area against the ratio of ECZ and MCZ nominal concentration. A good linear fit was found in the concentration range of 1–100 ng/mL. Correlation coefficients for all calibration curves were found to be superior to 0.99.

Limit of detection and limit of quantification

The lowest concentration of ECZ detected (LOD) and lowest limit of ECZ quantified (LOQ) were found to be 0.7 ng/mL and 2.0 ng/mL, respectively.

Precision and accuracy

Intra- and inter-day precision and accuracy was assessed using 2, 5, 10, 20, 50, 100 ng/mL ECZ solutions in acetonitrile/water (80/20) skin extract. Table S1 shows a summary of intra-day accuracy and precision and Table S2 shows a summary of inter-day accuracy and precision for the method.

Table 1. Intra-day precision and accuracy for quantification of ECZ in skin sample (values are given as mean ± SD).

[ECZ]	Intra-Day 1			Intra-Day 2			Intra-Day 3		
Theo (ng/mL)	[ECZ] Meas (ng/mL)	RSD (%)	Recovery (%)	[ECZ] Meas (ng/mL)	RSD (%)	Recovery (%)	[ECZ] Meas (ng/mL)	RSD (%)	Recovery (%)
2	2.4 ± 0.3	11.2	119.6	2.1 ± 0.0	1.2	107.1	2.1 ± 0.1	5.1	105.5
5	5.5 ± 0.2	3.6	111.0	5.5 ± 0.3	4.7	110.7	5.6 ± 0.1	2.0	111.6
10	10.8 ± 0.3	2.5	108.1	11.4 ± 0.3	2.9	114.2	10.9 ± 0.0	0.2	109.5
20	21.5 ± 0.2	2.5	107.6	21.2 ± 0.1	0.4	106.1	20.8 ± 0.3	1.6	103.9
50	53.5 ± 0.6	1.1	107.0	53.9 ± 0.8	1.5	107.9	51.5 ± 1.0	2.0	103.0
100	109.5 ± 0.4	0.4	109.5	107.3 ± 2.0	1.9	107.3	102.8 ± 1.4	1.4	102.8

Table 2. Inter-day precision and accuracy for quantification of ECZ in skin sample (values are given as mean ± SD).

[ECZ]	Intra-Day 1			Intra-Day 2			Intra-Day 3		
Theo (ng/mL)	[ECZ] Meas (ng/mL)	RSD (%)	Recovery (%)	[ECZ] Meas (ng/mL)	RSD (%)	Recovery (%)	[ECZ] Meas (ng/mL)	RSD (%)	Recovery (%)
2	2.1 ± 0.0	2.0	103.9	2.2 ± 0.2	10.6	112.0	2.3 ± 0.2	9.6	116.3
5	5.4 ± 0.1	2.0	104.1	5.6 ± 0.1	1.4	107.6	5.7 ± 0.1	1.0	109.9
10	10.8 ± 0.3	2.5	108.3	11.1 ± 0.4	3.4	111.0	11.2 ± 0.4	3.4	112.4
20	21.1 ± 0.7	3.3	105.6	21.2 ± 0.3	1.3	105.8	21.2 ± 0.2	0.7	106.2
50	52.6 ± 2.1	4.1	105.3	52.8 ± 0.9	1.6	105.6	53.5 ± 1.0	1.9	107.0
100	106.3 ± 4.4	4.1	106.3	107.0 ± 3.4	3.2	107.0	106.2 ± 3.1	2.9	106.2

The mean recovery observed for intra-day measurement on day 1 was from 107.0 to 119.6% (RSD 0.4–11.2%), on day 2 it was from 106.1 to 114.2% (RSD 0.4–4.7%) and on day 3 from 102.8 to 111.6% (RSD 0.2–5.1%). The mean recovery observed for inter-day 1 analysis was between 103.9 and 108.3% (RSD 2.0–4.1%), for inter-day 2 it was between 105.6 and 112.0% (RSD 1.3–10.6%), for inter-day 3 it was between 106.2 and 116.3% (RSD 0.7–9.6%).

The method was considered as accurate and precise as all measured values were within the acceptance limits of the ICH guidelines Validation of Analytical Procedure Q2(R1) (2005).

Abbreviations

Table S3 shows the abbreviations used in the article.

Table 3. Table of abbreviations.

AUC	area under the curve
BE	bioequivalence
CI	confidence interval
C_{max}	peak drug concentration in the blood
ECZ	econazole
EMA	European Medicines Agency
ICH	International Council for Harmonisation
IVPT	*in vitro* permeation test
IVRT	*in vitro* release test
LOD	limit of detection
LOQ	limit of quantification
MCZ	miconazole
MRM	multiple reaction monitoring
MS	mass spectrometry
OECD	Organisation for Economic Co-operation and Development
RMP	reference medicinal product
RSD	relative standard deviation
SC	*stratum corneum*
T_{max}	time at C_{max}
UHPLC	ultra-high pressure liquid chromotography
